# *Bifidobacterium animalis* subsp. *lactis* A6 Enhances Fatty Acid β-Oxidation of Adipose Tissue to Ameliorate the Development of Obesity in Mice

**DOI:** 10.3390/nu14030598

**Published:** 2022-01-29

**Authors:** Yanxiong Huo, Guoping Zhao, Jinwang Li, Ran Wang, Fazheng Ren, Yixuan Li, Xiaoyu Wang

**Affiliations:** 1 Key Laboratory of Precision Nutrition and Food Quality, College of Food Science & Nutritional Engineering, China Agricultural University, Beijing 100083, China; huoyanxiong195615@163.com (Y.H.); renfazheng@cau.edu.cn (F.R.); liyixuan@cau.edu.cn (Y.L.); 2 School of Food and Health, Beijing Technology and Business University, Beijing 100048, China; zhaoguoping525@126.com (G.Z.); sdlijinwang@sina.com (J.L.); 3 Key Laboratory of Functional Dairy, Co-Constructed by Ministry of Education and Beijing Municipality, Department of Nutrition and Health, China Agricultural University, Beijing 100083, China; wangran@cau.edu.cn

**Keywords:** *Bifidobacterium animalis* subsp. *lactis* A6, obesity, fatty acid β-oxidation, acetate, GPR43, PPARα

## Abstract

Fatty acid β-oxidation (FAO) is confirmed to be impaired in obesity, especially in adipose tissues. We previously proved that *Bifidobacterium animalis* subsp. *lactis* A6 (BAA6) had protective effects against diet-induced obesity. However, whether BAA6 enhances FAO to ameliorate the development of obesity has not been explored. After being fed with high-fat diet (HFD) for 9 weeks, male C57BL/6J mice were fed HFD or BAA6 for 8 weeks. In vitro study was carried out using 3T3-L1 adipocytes to determine the effect of BAA6 culture supernatant (BAA6-CM). Here, we showed that administration of BAA6 to mice fed with HFD decreased body weight gain (by 5.03 g) and significantly up-regulated FAO in epididymal adipose tissues. In parallel, FAO in 3T3-L1 cells was increased after BAA6-CM treatment. Acetate was identified as a constituent of BAA6-CM that showed a similar effect to BAA6-CM. Furthermore, acetate treatment activated the GPR43-PPARα signaling, thereby promoting FAO in 3T3-L1 cells. The levels of acetate were also elevated in serum and feces (by 1.92- and 2.27-fold) of HFD-fed mice following BAA6 administration. The expression levels of GPR43 and PPARα were increased by 55.45% and 69.84% after BAA6 supplement in the epididymal fat of mice. Together, these data reveal that BAA6 promotes FAO of adipose tissues through the GPR43-PPARα signaling, mainly by increasing acetate levels, leading to alleviating the development of obesity.

## 1. Introduction

Obesity is a common health problem for individuals today, which could increase the risk of metabolic diseases [1]. It is characterized by elevating fat mass through imbalance of anabolism and catabolism, especially fatty acid β-oxidation (FAO) processes in adipocytes [2]. FAO is shown to be low in obesity, and lean persons have high FAO [3]. Thus, the reduction in FAO has been supposed to be a key driver of the lipid accumulation in obese individuals [4].

FAO is recognized as an essential factor in obesity [5,6]. It has been shown that obese patients have decreased FAO protein levels in adipose tissues [7]. A large body of evidence has shown that up-regulating the expression of FAO-related proteins could improve the obese phenotype [8]. Others have demonstrated that overexpression of FAO-related proteins, such as carnitine palmitoyltransferase-1 (CPT1), in adipocytes could decrease triglyceride (TG) accumulation in obesity [9]. Currently, approaches of drug and phytochemical modulation are used to promote FAO in obesity. Several drugs, such as rimonabant, have been used towards weight management by enhancing FAO [10]. However, the high cost and potential side effects for this kind of drug causes dissatisfaction to patients [11]. Although, phytochemicals such as phenolic compounds, polyunsaturated fatty acids, and plant sterols could also increase FAO [11]. Their applications are limited by weak stability [12]. Recently, probiotics are thought to possess various beneficial effects on human health with great safety [13,14]. More and more research has proved the beneficial effects of probiotics on enhancement of FAO in obesity, and this strategy has been regarded as a natural therapeutic agent to control metabolic syndrome [15]. Indeed, many reports reveal that *Akkermansia muciniphila* (AKK) could increase FAO to ameliorate obesity [16], which has been used as a uniquely promising strain for its high therapeutic effects [17]. Several findings have shown that administration of probiotic bacteria, such as *Lactobacillus* genus, to a high-fat diet (HFD)-fed mice promotes FAO in fat tissues, thereby protecting against obesity [18,19]. Further, probiotics such as *Lactobacillus* genus have been shown to be able to reduce fat percentage in obese people [20]. Notably, probiotics treatment to enhance FAO is considered as a means to alleviate obesity. However, the mechanism by which probiotics increase FAO has not been fully clarified.

G protein-coupled receptors (GPRs) play pivotal roles in cell signaling to maintain energy homeostasis [21,22]. They are known to regulate various adipocyte functions, including FAO and thermogenesis [23]. GPR43 is a key member for GPRs which is involved in adipocyte lipid metabolism [24,25]. Many studies have reported that GPR43 might up-regulate FAO-related genes expression [26]. Besides, peroxisomal proliferator-activated receptor α (PPARα) has a master function in the regulation of FAO [27]. Some findings have pointed out that PPARα activation might enhance gene expression of fatty acid-metabolizing proteins to promote FAO in adipose tissues [28]. Conversely, inhibition of PPARα expression could reduce FAO in adipocytes [29]. Hence, GPR43-PPARα signaling pathway is crucial to regulate FAO.

*Bifidobacterium animalis* subsp. *lactis* A6 (BAA6) is a probiotic which can stay active under gastric juice environment [30]. Additionally, our previous research suggested that administration of BAA6 to obese mice could reduce body weight gain [31]. However, the mechanism of BAA6 to ameliorate the development of obesity has not been clarified. Thus, the objective of this research was to test the hypothesis that BAA6 could promote FAO of adipose tissues to ameliorate the development of obesity through GPR43-PPARα signaling.

## 2. Materials and Methods

### 2.1. Preparation of Bacterial Cultures, BAA6-CM and Dead BAA6

BAA6 (CGMCC No. 9273) was obtained from the feces of a centenarian (Bama, Guangxi, China). BAA6 was incubated in none oxygen conditions, as described in previous study [31].

AKK (JCM 30893) was provided by Japan Collection of Microorganisms RIKEN BioResource Research Center (Ibaraki, Japan) and incubated in none oxygen condition, as presented previously [31].

BAA6 culture supernatant (BAA6-CM) was obtained as with the previous method [32]. Dead BAA6 was prepared as previously reported [33]. Briefly, BAA6 cells, grown to 10^9^ colony-forming units (CFU)/mL, were centrifugated at 3000× *g* at 4 °C for 15 min. BAA6-CM was obtained from the cell-free supernatant. Then, the collected BAA6 cells were washed twice and reconstituted by physiological saline. Plate count was used to measure bacterial concentration. The dead BAA6 was prepared at 80 °C for 30 min.

### 2.2. Animals and Experimental Design

Male C57BL/6J mice (4-week-old, Body weight 18.63 ± 1.02 g) were provided by Beijing HFK Bioscience Co. Ltd. (Beijing, China). They were raised under a stable temperature (23 ± 1 °C) and 12 h light/dark cycle for 1 week. Mice were randomly assigned into two groups: normal diet (ND group, *n* = 6) and HFD (*n* = 22) for 9 weeks. Composition of diets was shown in Supplementary Table S1. Then, mice fed HFD (Body weight 30.62 ± 1.21 g) were randomly divided into three groups (*n* = 6): (1) HFD group; (2) HFD + BAA6 group, gavage of BAA6 at 10^9^ CFU/kg per day for another 8 weeks; (3) HFD + AKK group (positive group), gavage of AKK at 10^9^ CFU/kg per day for another 8 weeks. Mice were orally administrated with 0.9% saline solution in ND and HFD groups. Body weight and food intake were measured every week. When the experiment ended, mice were fasted for 12 h and then anesthetized by CO_2_. Blood and fat tissues were collected. The fat tissues and serum were preserved at −80 °C for further detection. The experimental processes were approved by the Animal Experimentation Committee of China Agricultural University (AW18080202-1).

### 2.3. Cell Differentiation

The 3T3-L1 preadipocytes were obtained from American Type Culture Collection (Manassas, VA, USA). Cells were grown in six-well plates (2 × 10^5^ cells/well) with Dulbecco’s modified Eagle’s medium (DMEM), containing 10% fetal bovine serum (FBS, Gibco Life Technologies, Rockville, MD, USA) at 37 °C and incubated to confluence. Subsequently, cells were differentiated, employing DMEM with FBS (10%), insulin (2 µg/mL), 3-isobutyl-1-methylxanthine (0.5 mmol/L), and dexamethasone (0.25 µmol/L) for 3 days. They continued incubation with insulin for another 5 days to fully differentiate into adipocytes [34].

### 2.4. Cell Viability Assay

The 3T3-L1 cell viability was detected by cell counting kit-8 assay (Beyotime Biotechnology, Beijing, China) according to the manufacturer’s instructions. In brief, 3T3-L1 preadipocytes were grown in 96-well plates (1 × 10^4^ cells/well). Subsequently, cells were differentiated into adipocytes. They were treated with BAA6-CM (0, 0.5, 1, 2, 4, and 8%) for 1–5 days and dead BAA6 (0, 10^4^, 10^5^, 10^6^, 10^7^, and 10^8^ cells/mL) for 1–4 days, respectively. After treatment, CCK8 reagent was added and incubated for 1 h at 37 °C. Microplate reader (Bio-Rad, Hercules, CA, USA) was used to assess cell viability (at 450 nm) [35].

### 2.5. Biochemical Assay of Serum

Total cholesterol (TC), TG, and high-density lipoprotein cholesterol (HDL-C) were analyzed by No. 3 Hospital of Beijing University [36].

### 2.6. Metabolic Assessment

Body fat was assessed by Body Composition Analysis (MiniQMR23-060H-I, Shanghai Niumag Corporation, Shanghai, China). Oxygen consumption and respiratory exchange ratio (RER) were measured through mouse Comprehensive Laboratory Animal Monitoring System metabolic cages [34].

### 2.7. TG Quantification Assay in 3T3-L1 Cells

TG concentration was detected through commercial TG assay kit (Nanjing Jiancheng Bioengineering Institute, Nanjing, China) as reported previously [37].

### 2.8. Hematoxylin and Eosin and Oil Red O Staining

Hematoxylin and eosin assay, in epididymal adipose tissues, was performed as with the previous method [34]. Oil Red O (ORO) assay, in 3T3-L1 cells, was conducted as previously described [38]. Images were obtained through a fluorescent inverted microscope (DMi8, Leica, Weztlar, Germany) and analyzed by a blind observer using Image Pro Plus A6 (version 6.0.0.260, Media Cybernetics Corporation, Las Vegas, NV, USA).

### 2.9. β Oxidation Study in 3T3-L1 Cells

β oxidation assessment was estimated through a Seahorse Bioscience XF96 Analyzer (Seahorse Bioscience Inc., MA, USA), as described previously [39]. Briefly, oxygen consumption rate (OCR) was analyzed in FAO Assay Medium, followed by treatment with or without etomoxir (45 mmol/L). Then, 25 mmol/L XF bovine serum albumin (BSA) or 167 mmol/L XF Palmitate-BSA (#102720-100, Agilent Technologies, Wilmington, DE, USA) was added in response to oligomycin (1.2 mmol/L), fluoro-carbonyl cyanide phenylhydrazone (1 mmol/L), and rotenone/antimycin A (1 mmol/L) (#101706-100, Agilent Technologies, Wilmington, DE, USA) based on recommending processes. β oxidation was calculated according to Timper et al.’s protocol [39].

### 2.10. Analysis for Acetate in BAA6-CM, Serum and Feces

Acetate was detected by GC-MS/MS. Wuhan MetWare Biotechnology was in charge of extracting the sample and quantifying metabolites, as previously described [40]. Briefly, samples of BAA6-CM, serum, and feces were thawed and vortexed for 1 min prior to analysis. Samples (50 μL) were added to 100 μL of 36% phosphoric acid solution. The mixture was vortexed for 3 min and 150 μL methyl tert-butyl ether (containing 2 μg/mL 2-methylvaleric acid as an internal standard) solution was added. After that, centrifugal force at 12,000× *g* was used to centrifuge the mixture at 4 °C for 10 min. The supernatant was used for further analysis. Agilent 7890B instrument, coupled to a 7000D Triple Quadrupole gas chromatography mass spectrometry system with a DB-FFAP column (30 m × 0.25 mm × 0.25 μm, J&W Scientific, Folsom, CA, USA), was utilized. The carrier gas was helium, and the flow rate was 1.2 mL/min. The oven temperature was held at 90 °C for 1 min, risen to 100 °C at a rate of 25 °C/min, risen to 150 °C at a rate of 20 °C/min, held on 0.6 min, risen to 200 °C at a rate of 25 °C/min, and kept for 0.5 min after running for 3 min. The quantitation of acetate in BAA6-CM, serum, and feces was counted using constructing calibration curves.

### 2.11. Analysis of Intracellular Calcium Concentration in 3T3-L1 Cells

The content of intracellular calcium (Ca^2+^) was measured as previously reported [41]. In brief, 3T3-L1 cells were cultured in 24-well plates (1 × 10^5^ cells/well). Then, cells were treated with DMEM, including 5 μmol/L Fura-2/AM (Beyotime Biotechnology, Beijing, China) for 45 min at 37 °C. Then, the 3T3-L1 cells were incubated with phosphate-buffered saline containing BSA (0.2%) for 5 min. The ratio of fluorescence intensities was assessed by changing excitation wavelengths of between 340 and 380 nm, with emission at 510 nm. The concentration was calculated as in the previous study [41].

### 2.12. Western Blot Analysis

Western blots were carried out as previously described [31]. Image J software (version 2.0, Microsoft Corporation, Redmond, WA, USA) was used to assess the band signal intensities [42]. Primary antibodies were listed as follows: fatty acid synthase (FAS, 3189s), phosphorylated acetyl CoA carboxylase (p-ACC, 3661s), ACC (3676s), hormone-sensitive lipase (HSL, 4107s), adipose triglyceride lipase (ATGL, 2138s), AMP-activated protein kinase (AMPK, 2532s) and phosphorylated AMPK (p-AMPK, 2535s) antibodies (Cell Signaling Technology, Boston, MA, USA). Carnitine palmitoyltransferase-2 (CPT2, 26555-1-AP) and PPARα (66826-1-lg) antibodies were provided from Proteintech (Rosemont, IL, USA). CPT1 (YN3388), long-chain acyl-CoA dehydrogenase (ACADL, YT6498), medium-chain acyl-CoA dehydrogenase (ACADM, YT5024), calcium/calmodulin-dependent protein kinase β (CAMKKβ, YT0624), phosph-CAMKKβ (p-CAMKKβ, YP1285), and GPR41 (YT2020) antibodies were from Immunoway (Plano, TX, USA). GPR43 (GTX00823) antibody was obtained from Gene Tex (San Antonio, CA, USA). β-Actin (bs-10966R) antibody was provided by Bioss (Beijing, China).

### 2.13. PPARα and GPR43 Knockdown

PPARα or GPR43-specific small interfering RNA (siRNA) was used to transfect cells by Lipofectamine 2000 (Thermo Fisher Scientific, Waltham, MA, USA) (Table S2, Supplementary Materials), according to the manufacturer’s instruction.

### 2.14. Statistical Analyses

All values were shown as mean ± standard deviation (SD). SPSS 21.0 (SPSS Inc., Chicago, IL, USA) was used to analyze data. The Shapiro–Wilk test was used to assess Gaussian distribution. Equal variance was analyzed by Levene’s test. Statistical comparison was measured by Tukey’s post-test after ANOVA analysis. Differences were considered significant when *p* < 0.05.

## 3. Results

### 3.1. BAA6 Decelerated Body Weight Gain and Lipid Accumulation in Obese Mice

**Figure 1:**
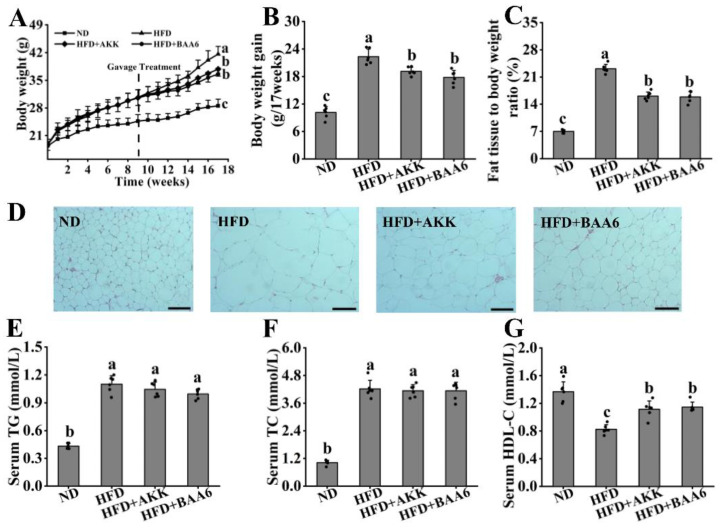
Effect of BAA6 administration on metabolic characteristics in high-fat diet (HFD)-fed mice. (**A**) Body weight, (**B**) body weight gain, (**C**) relative adipose tissue, (**D**) representative hematoxylin and eosin staining in epididymal adipose tissues (scale bar, 200 μm), serum levels of (**E**) triglyceride (TG), (**F**) total cholesterol (TC), and (**G**) high-density lipoprotein cholesterol (HDL-C) were analyzed. Values are shown as means ± SD (*n* = 6). Different lowercase letters mean remarkable differences between groups at *p* < 0.05. ND, fed normal diet; HFD, fed high-fat diet; HFD + BAA6, fed high-fat diet and administrated with *Bifidobacterium animalis* subsp. *lactis* A6 (BAA6); HFD + AKK, fed high-fat diet and administrated with *Akkermansia muciniphila* (AKK).

To investigate the effect of BAA6 in alleviating the development of obesity in mice, the levels of body weight, food intake, fat mass, and serum lipid profiles of mice were measured. There was no remarkable difference in food intake of all mice (Table S3, Supplementary Materials). In HFD group, the body weight gain, final body weight, and relative fat weight were higher than ND group, which were reduced after gavage of BAA6 and AKK (positive control) (Figure 1A–C). Furthermore, the number of adipocytes increased, and the cell area reduced after gavage with BAA6 and AKK, compared to HFD group (Figure 1D). Meanwhile, HFD group displayed higher concentration of serum TG and TC, as well as lower concentrations of HDL-C than ND group (Figure 1E–G). Importantly, BAA6 and AKK treatment markedly increased HDL-C levels. Together, the above data indicated that BAA6 treatment could decrease fat mass and body weight, and BAA6 showed similar effects to AKK.

### 3.2. BAA6 Enhanced FAO in Adipose Tissues of Obese Mice

**Figure 2:**
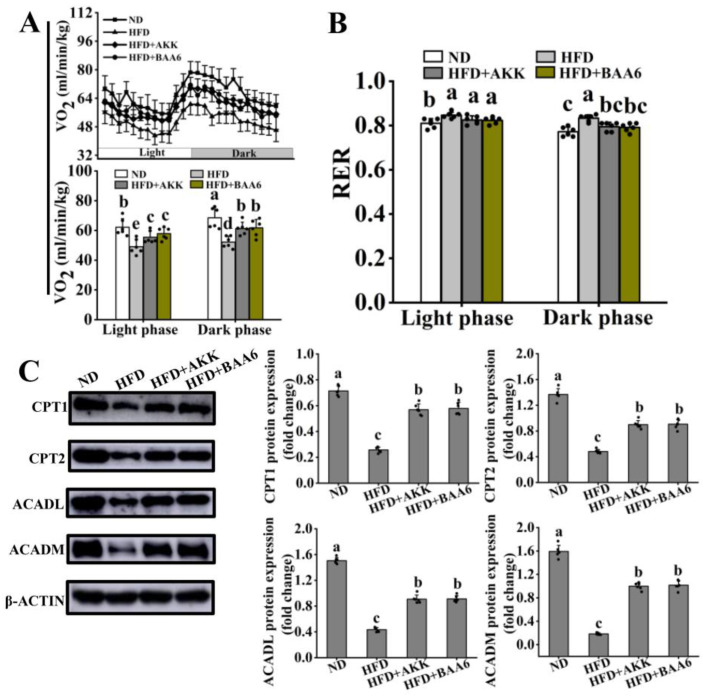
Effect of BAA6 on fatty acid β-oxidation (FAO) for epididymal adipose tissues in HFD-fed mice. (**A**) O_2_ consumption and (**B**) respiratory exchange ratio (RER) were detected during a 12 h light/dark cycle in mice. (**C**) Expression levels of CPT1, CPT2, ACADL, and ACADM in epididymal adipose tissues were measured. Values are shown as means ± SD (*n* = 6). Different lowercase letters mean remarkable differences between groups at *p* < 0.05. CPT1, carnitine palmitoyltransferase-1; CPT2, carnitine palmitoyltransferase-2; ACADL, long-chain acyl-CoA dehydrogenase; ACADM, medium-chain acyl-CoA dehydrogenase.

A great deal of research proved that low FAO contributed to obesity. The efficacy of BAA6, in alleviating the development of obesity, has been confirmed. Thus, the possible role of FAO was further explored. To investigate whether BAA6 alleviating the development of obesity was associated with enhancement of FAO in fat tissues, we firstly assessed the energy expenditure and FAO of obese mice in the resting state. Compared to ND group, energy expenditure (indicated by decreased oxygen consumption, VO_2_) reduced in HFD group in both light and dark phases, while it was increased by BAA6 and AKK treatments (Figure 2A). Moreover, there was a remarkable decrease in FAO (indicated by increased RER) in HFD group during light and dark phases compared with ND group, which was recovered by BAA6 and AKK treatments during dark cycle (Figure 2B). Subsequently, proteins associated with lipid metabolism in the epididymal adipose tissues were measured. Western blotting of epididymal adipose tissues revealed the similar levels of lipid synthesis-related proteins, such as FAS and p-ACC, in BAA6 and AKK-treated mice relative to HFD mice, while lipid catabolism-related protein (HSL and ATGL) levels were significantly higher, after BAA6 and AKK supplementations, compared with HFD group (Figure S1, Supplementary Materials). Further, FAO-related proteins were assessed in the epididymal adipose tissues. The FAO-related protein levels (CPT1, CPT2, ACADL, and ACADM) in adipose tissues of HFD mice were lower than ND mice (Figure 2C), whereas these protein levels were increased, after BAA6 and AKK treatments, compared to HFD group. Our results indicated that BAA6 could enhance FAO in adipose tissues to alleviate the development of obesity.

### 3.3. BAA6-CM Promoted FAO in 3T3-L1 Adipocytes

**Figure 3:**
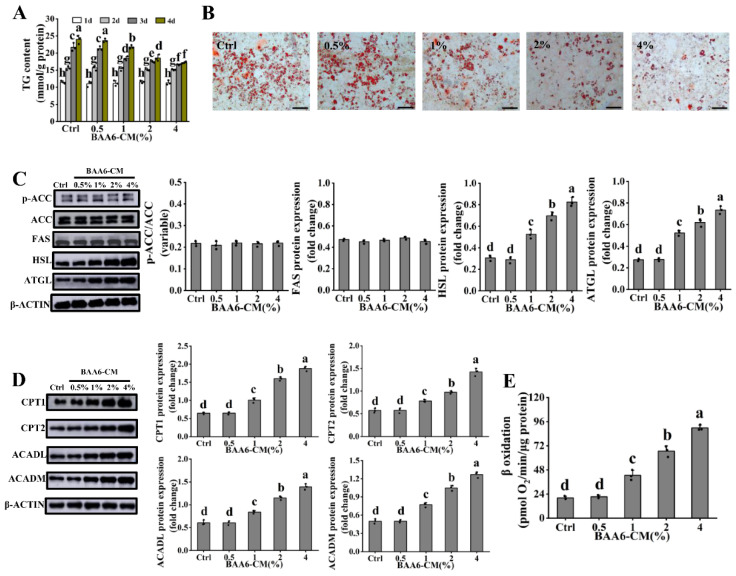
Effect of BAA6 culture supernatant (BAA6-CM) on TG content and FAO in 3T3-L1 cells. (**A**) TG concentration, in the presence of BAA6-CM (0–4%), for 1–4 days. (**B**) Photograph of Oil Red O (ORO) staining (scale bar, 200 μm) and western blot analysis, showing the levels of (**C**) p-ACC, FAS, HSL, and ATGL, and (**D**) levels of protein expression of CPT1, CPT2, ACADL, and ACADM after treatment with 0–4% BAA6-CM for 3 days. (**E**) β oxidation, measured by Seahorse XF platform, following various concentrations of 0–4% BAA6-CM for 3 days. Values are shown as means ± SD. Different lowercase letters mean remarkable differences between groups at *p* < 0.05.

To gain additional insights into the underlying mechanisms of BAA6 on FAO in adipose tissues, 3T3-L1 cells were chosen for further study. BAA6-CM and dead BAA6 were used to treat 3T3-L1 cells because it’s unlikely that live BAA6 could reach adipose tissues in vivo. 3T3-L1 cells were exposed to BAA6-CM at 0–4% for 1–4 days, and dead BAA6 at 0–10^7^ cells/mL for 1–3 days, to ensure cell viability (Figure S2A,B, Supplementary Materials). As shown in Figure 3A, no significant differences were observed in the TG content during various concentrations of BAA6-CM treatment for 1–2 days. However, TG content was markedly decreased with the increase concentration of BAA6-CM for 3–4 days, and there was no significant difference following BAA6-CM treatment between 3 and 4 days at 4% BAA6-CM. In addition, dead BAA6 treatment showed no remarkable effect on TG content (Figure S3, Supplementary Materials). Thus, BAA6-CM (0–4%) treatment for 3 days was used for further analyses. As visualized by ORO staining, the supplement of BAA6-CM diminished lipid accumulation of 3T3-L1 adipocytes in a concentration-dependent manner (Figure 3B). These data indicated that BAA6-CM had a positive effect on lipid metabolism in vitro.

To test the effects of FAO on TG reduction after BAA6-CM treatment, lipid metabolism-related proteins were firstly assessed. As displayed in Figure 3C, the lipid synthesis-related protein levels (FAS and p-ACC) had no remarkable change in BAA6-CM-treated cells relative to the control group. However, lipid catabolism-related protein (HSL and ATGL) contents were significantly higher in the BAA6-CM group in a concentration-dependent manner, indicating that BAA6-CM could promote lipid catabolism. Next, the key proteins in the processes of FAO were measured. BAA6-CM treatment significantly increased levels of FAO-related proteins (CPT1, CPT2, ACADL, and ACADM) by a dose-dependent way (Figure 3D). Further, FAO was evaluated using the Seahorse XF platform. As shown in Figure 3E, BAA6-CM treatment could significantly up-regulate FAO, compared to control group. These results implicated that BAA6-CM could promote FAO to decrease TG accumulation in vitro.

### 3.4. Acetate Is a Key BAA6-CM Metabolite That Increases FAO in 3T3-L1 Cells

**Figure 4:**
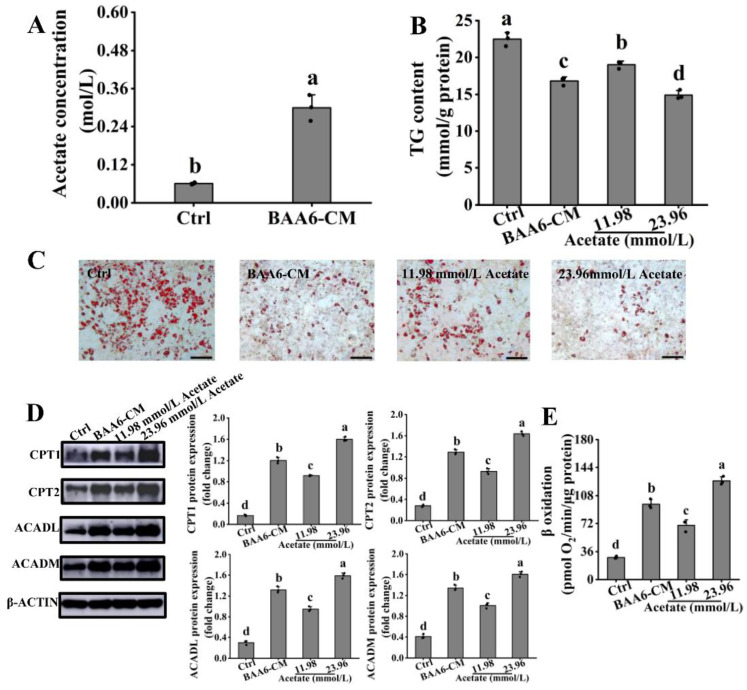
Acetate, as a key BAA6-CM metabolite, enhanced FAO in 3T3-L1 cells. (**A**) Acetate contents detected by GC-MS/MS in BAA6-CM. (**B**) TG levels after treatment with BAA6-CM (4%) or acetate (11.98 and 23.96 mmol/L; one and two times of acetate concentration in 4% BAA6-CM treatment) for 3 days. (**C**) Photograph of ORO staining (scale bar, 200 μm), and (**D**) levels of protein expression of CPT1, CPT2, ACADL, and ACADM after treatment with 4% BAA6-CM or acetate (11.98 and 23.96 mmol/L) for 3 days in 3T3-L1 cells. (**E**) β oxidation, measured by Seahorse XF platform, after treatment with 4% BAA6-CM or acetate (11.98 and 23.96 mmol/L) for 3 days. Values are shown as means ± SD. Different lowercase letters mean remarkable differences between groups at *p* < 0.05.

We then wondered which kind of bioactive component in BAA6-CM was responsible for enhancing FAO in 3T3-L1 cells. Interestingly, accumulating evidence suggested that acetate could been generated by genus *Bifidobacterium* [43], and it was proved to enhance FAO in vivo and in vitro [44]. We found that acetate levels in BAA6-CM was higher compared with control media (Figure 4A). Subsequently, the effect of acetate on TG content was tested in vitro. As displayed in Figure 4B, 11.98 mmol/L acetate treatment (acetate concentration in 4% BAA6-CM group) had lower TG content in cells than control group, which was about 62% of the function, as compared to BAA6-CM treatment. Acetate treatment (23.96 mmol/L; two times of acetate concentration in 4% BAA6-CM group) existed a higher effect on TG content than that of BAA6-CM treatment. As visualized by ORO staining, the supplement of BAA6-CM and acetate could remarkably diminish lipid accumulation in vitro, compared to the control group (Figure 4C).

To assess the effect of acetate on FAO, the levels of key proteins involved in FAO were firstly measured. Figure 4D showed that acetate and BAA6-CM treatment remarkably elevated the expression of CPT1, CPT2, ACADL, and ACADM proteins relative to control group. Then, FAO was assessed using the Seahorse XF platform after acetate treatment. As shown in Figure 4E, FAO was remarkably enhanced by acetate and BAA6-CM treatments. Meanwhile, acetate (23.96 mmol/L) treatment obviously promoted FAO compared to the BAA6-CM group. Thus, acetate (23.96 mmol/L) treatment was used for further analyses. These data indicated that acetate was essential for BAA6-CM to enhance FAO in vitro.

### 3.5. PPARα Signaling Mediated the Regulation of Acetate on FAO in 3T3-L1 Cells

**Figure 5:**
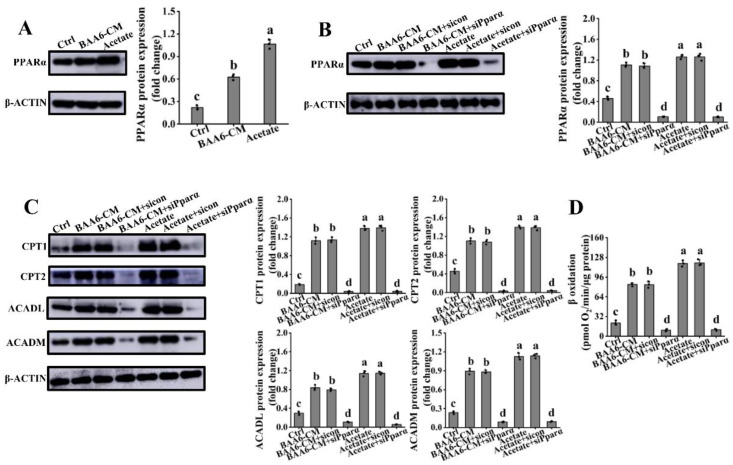
Effect of acetate or BAA6-CM on peroxisomal proliferator-activated receptor α (PPARα) signaling in 3T3-L1 cells. (**A**) Protein expression levels of PPARα after treatment with 4% BAA6-CM or 23.96 mmol/L acetate for 3 days. Levels of protein expression of (**B**) PPARα, and (**C**) CPT1, CPT2, ACADL, and ACADM after cell treated with control or PPARα-special small interfering RNA (siRNA), followed by addition of 4% BAA6-CM or 23.96 mmol/L acetate for 3 days. (**D**) β oxidation, measured by Seahorse XF platform after cell treatment, with control or PPARα-special siRNA, followed by addition of 4% BAA6-CM or 23.96 mmol/L acetate for 3 days. Values are shown as means ± SD. Different lowercase letters mean remarkable differences between groups at *p* < 0.05.

PPARα signaling has a master role in the regulation of FAO. Firstly, the expression of PPARα was investigated. The protein expression level of PPARα was significantly enhanced by acetate and BAA6-CM treatments, which was lower in the control group (Figure 5A). This data suggested that acetate could up-regulate PPARα expression. Then, to better clarify the role of PPARα in acetate and BAA6-CM-regulating FAO, a PPARα-specific siRNA was introduced into 3T3-L1 cells. The levels of PPARα expression were markedly declined after silencing treatment (Figure 5B). Moreover, acetate and BAA6-CM-induced increase in FAO key protein (CPT1, CPT2, ACADL, and ACADM) levels were diminished by PPARα knockdown (Figure 5C). In these experiments, the siRNA control group was not significantly different from the acetate and BAA6-only control, displaying no additional siRNA effects. What’s more, knockdown of PPARα also disturbed the BAA6-CM and acetate-induced up-regulation of FAO (Figure 5D). These results indicated that acetate enhanced FAO through modulating PPARα signaling.

**Figure 6:**
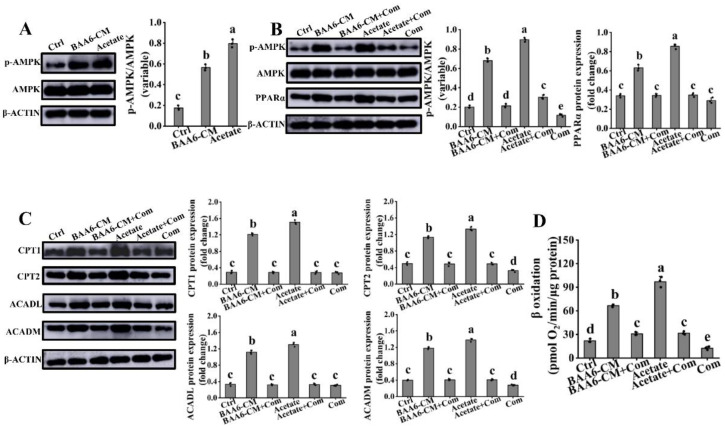
Effect of acetate or BAA6-CM on AMPK activity in 3T3-L1 cells. (**A**) Western blot analysis showing the levels of p-AMPK after treatment with 4% BAA6-CM or 23.96 mmol/L acetate for 3 days. Protein expression levels of (**B**) p-AMPK and PPARα and (**C**) CPT1, CPT2, ACADL, and ACADM in cells pretreated with 20 μmol/L compound C (Com), followed by treatment with 4% BAA6-CM or 23.96 mmol/L acetate for 3 days. (**D**) β oxidation, measured by Seahorse XF platform, after cells pretreatment with 20 μmol/L Com, followed by treatment with 4% BAA6-CM or 23.96 mmol/L acetate for 3 days. Values are shown as means ± SD. Different lowercase letters mean remarkable differences between groups at *p* < 0.05. AMPK, AMP-activated protein kinase; p-AMPK, phosphorylated AMPK.

Several pieces of evidence verified that PPARα was the downstream target of AMPK [45]. Therefore, to explore whether activation of PPARα signaling was related to AMPK, we firstly determined the effects of acetate and BAA6-CM on AMPK activity in 3T3-L1 cells. Acetate and BAA6-CM markedly improved the p-AMPK protein expression, compared to control group (Figure 6A). Then, when cells were pretreated with compound C (Com, an AMPK inhibitor), the levels of p-AMPK protein expression were markedly reduced (Figure 6B). Similarly, the up-regulation of acetate and BAA6-CM on PPARα, CPT1, CPT2, ACADL, and ACADM protein levels were markedly abrogated after the addition of Com (Figure 6B,C). Further, the increase in FAO for acetate and BAA6-CM was significantly destroyed following pretreatment with Com (Figure 6D). These findings revealed that AMPK played an important role in modulating PPARα signaling for acetate-mediated FAO in vitro.

### 3.6. Acetate Activated GPR43 in 3T3-L1 Cells

**Figure 7:**
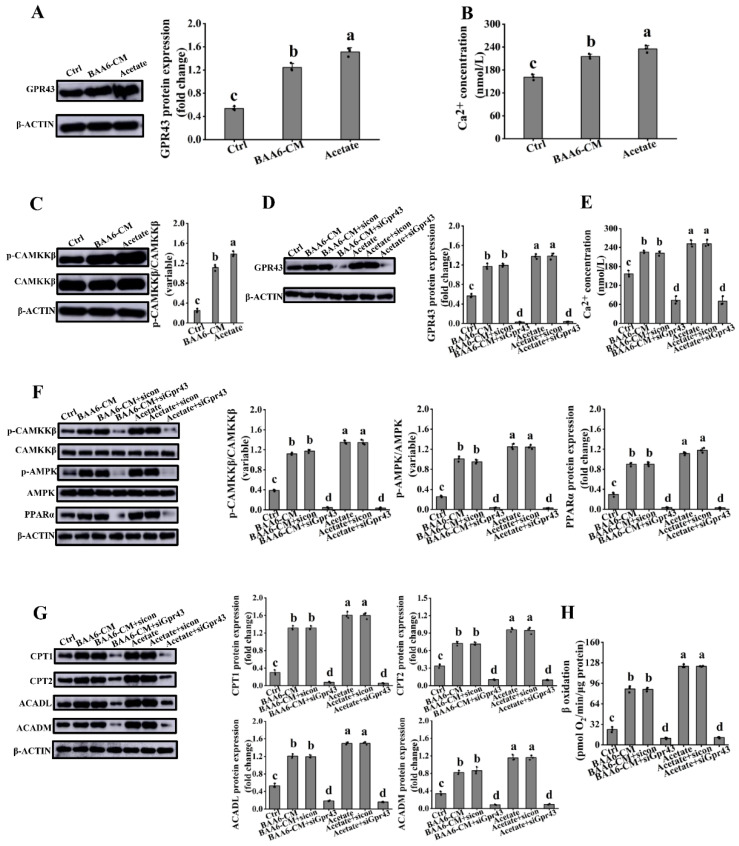
Effect of acetate or BAA6-CM on GPR43 signaling in 3T3-L1 cells. Western blot analysis showing the levels of (**A**) GPR43, (**B**) Ca^2+^ contents, and (**C**) p-CAMKKβ in 3T3-L1 cells after treatment with 4% BAA6-CM or 23.96 mmol/L acetate for 3 days. Protein expression levels of (**D**) GPR43 and (**E**) Ca^2+^ concentration, after the cell is treated with control or GPR43-special siRNA, followed by addition of 4% BAA6-CM or 23.96 mmol/L acetate for 3 days. (**F**) Protein expression levels of p-CAMKKβ, p-AMPK, PPARα, (**G**) protein levels of CPT1, CPT2, ACADL, ACADM, and (**H**) β oxidation after the cell is treated with control or GPR43-special siRNA, followed by addition of 4% BAA6-CM or 23.96 mmol/L acetate for 3 days. Values are shown as means ± SD. Different lowercase letters mean remarkable differences between groups at *p* < 0.05. GPR43, G protein-coupled receptor 43; p-CAMKKβ, calcium/calmodulin-dependent protein kinase β phosphorylation.

GPRs had a pivotal role in cell signaling, which could regulate energy homeostasis [21]. GPR43 and GPR41 are important members of GPRs, which are receptors of acetate [46]. Therefore, we investigated which receptor was activated following acetate and BAA6-CM treatments. GPR43 protein expression was markedly increased in adipocytes after a supplement of acetate and BAA6-CM, compared with control group (Figure 7A). However, there was no significant difference on GPR41 expression after acetate and BAA6-CM treatments, compared to control group (Figure S4, Supplementary Materials). These results showed that acetate and BAA6-CM could activate GPR43. In addition, GPR43 might regulate AMPK activity by Ca^2+^/CAMKKβ [47]. Thus, the concentration of intracellular Ca^2+^ and p-CAMKKβ protein expression level were further measured. In the presence of acetate and BAA6-CM, the levels of Ca^2+^ and p-CAMKKβ protein were remarkably increased, compared with control group (Figure 7B,C).

To verify whether acetate actually activated GPR43 to elicit downstream signals, the expression of GPR43 was silenced using GPR43 siRNA. Results showed that GPR43 siRNA was effective in decreasing the expression of GPR43 (Figure 7D). Consistently, acetate and BAA6-CM-induced up-regulation levels of intracellular Ca^2+^, p-CAMKKβ, p-AMPK, and PPARα proteins were blunted by GPR43 knockdown (Figure 7E,F). Moreover, the acetate and BAA6-CM-stimulated expression of proteins (CPT1, CPT2, ACADL, and ACADM) and enhancement of FAO were also destroyed by the knockdown of GPR43 (Figure 7G,H). Hence, these data indicated that activation of GPR43 signaling, by acetate, was crucial to regulate FAO in vitro.

### 3.7. BAA6 Activated GPR43-PPARα Signaling In Vivo

**Figure 8:**
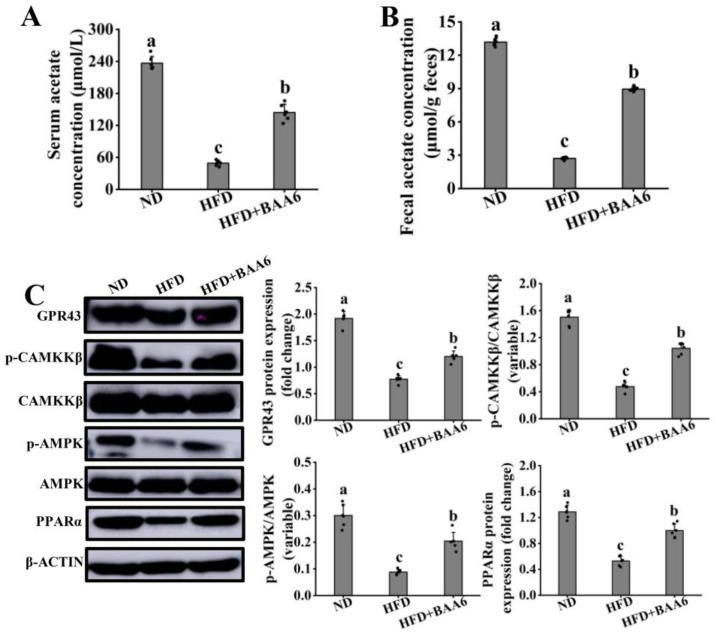
Effect of BAA6 administration on GPR43-PPARα signaling of epididymal adipose tissues in HFD-fed mice. Acetate concentration in serum (**A**) and feces (**B**). (**C**) Western blot results of GPR43, p-CAMKKβ, p-AMPK, and PPARα, following daily treatment with 10^9^ CFU/kg of BAA6. Values are shown as means ± SD (*n* = 6). Different lowercase letters mean remarkable differences between groups at *p* < 0.05.

To determine whether GPR43-PPARα signaling was activated by BAA6 in adipose tissues of obese mice, we firstly explored the acetate contents in serum and feces. HFD mice had lower acetate concentration in serum and feces compared to ND mice. Meanwhile, levels of acetate in the HFD group were also lower than HFD + BAA6 group (Figure 8A,B). These results suggested that the raise of acetate level was due to BAA6. Then, we investigated the expression of GPR43, p-CAMKKβ, p-AMPK, and PPARα proteins. Results showed that HFD mice had lower levels of GPR43, p-CAMKKβ, p-AMPK, and PPARα proteins relative to ND mice. Compared with HFD group, BAA6 treatment of mice could remarkably up-regulate the expression of GPR43, p-CAMKKβ, p-AMPK, and PPARα proteins (Figure 8C). These findings suggested that GPR43-PPARα pathways in adipose tissues were also activated by BAA6 treatment in vivo.

## 4. Discussion

Given abundant research indicating the positive influences for probiotics in the management of obesity, this approach is attracting more and more attention [15,48]. Our results showed that BAA6 treatment had a similar effect in alleviating the development of obesity to AKK, which was verified to ameliorate obesity in many studies [49]. Thus, in our study, we investigated the mechanism of BAA6 to alleviate the development of obesity.

It was reported that O_2_ expenditure tended to decrease in HFD-fed mice, which exhibited a high RER [50]. Elevated RER was a characteristic of weight gain in obesity [51]. Meanwhile, RER was an indicator for FAO in obese mice, and low RER reflected the enhancement of FAO [52]. Thus, FAO could be a vital means to manage obesity [6]. In this study, these results suggested that BAA6 could remarkably increase O_2_ expenditure and reduce RER during the dark cycle, which meant that BAA6 treatment was beneficial to enhance FAO in obese mice. Some studies have demonstrated that FAO in obesity is impaired, particularly in adipose tissues [53,54]. Adipose tissue is regarded as the largest organ of the body [55]. In lean individuals, adipose tissue accounted for 10% of body weight, but the proportion could increase up to 40% in obese subjects [55]. Meanwhile, FAO is one of the major biochemical factors to maintain function of fat tissues [56]. Unfortunately, the levels of CPT1, CPT2, ACADL, and ACADM proteins were decreased in the adipose tissues of obese mice, which were the major modulators for FAO [57]. A series of studies found that administration of probiotic bacteria, such as *Lactobacillus* genus, to HFD-fed mice promoted FAO in adipose tissues [18,19]. In addition, *Lactobacillus acidophilus* NS1 could enhance the FAO-related proteins expression and then, reduce body fat accumulation [58]. Our study also showed that the levels of CPT1, CPT2, ACADL, and ACADM proteins, in epididymal adipose tissues, were elevated (by 121.06%, 87.88%, 109.71%, and 452.62%, respectively) after BAA6 treatment. Above findings revealed that FAO, in the epididymal adipose tissues for obese mice, could be remarkably enhanced by BAA6.

To gain additional insights into the underlying mechanisms of BAA6 on FAO in adipose tissues, 3T3-L1 cells, as common models in vitro, were chosen for further study [59]. It might be unlikely for live probiotics to directly arrive to the adipose tissue in vivo [60]. In that case, the components of dead cells or metabolites might be used for adipocytes [60]. We observed that TG content in 3T3-L1 cells was significantly decreased after BAA6-CM supplementation, which was not remarkably different after dead BAA6 treatment. These phenomena indicated that BAA6-CM had positive roles on lipid metabolism in vitro. Some researchers found that culture supernatant of *Bifidobacterium* and *Lactobacillus* genera induced the expression of FAO-related proteins in cells [61]. Thus, probiotic metabolites were important factors for regulating FAO. Our study showed that the key proteins expression in FAO was significantly increased under BAA6-CM treatment in vitro. Furthermore, results from Seahorse XF platform also indicated that FAO was increased (by 1.11-, 2.30-, and 3.44- fold) after different concentration of BAA6-CM treatment. According to these findings, we assumed that BAA6-CM treatment could promote FAO in adipocytes.

However, it was necessary to confirm which bioactive components produced by BAA6 could enhance FAO. Many studies found that probiotics could generate short-chain fatty acids (SCFAs), which had positive effects on the host [62]. Acetate is the most abundant SCFA in humans, accounting for 50% of total SCFAs [63]. Accumulating studies suggested that acetate could be produced by genus *Bifidobacterium* [43]. Similarly, we found that the level of acetate in BAA6-CM was enhanced by 3.92-fold. Meanwhile, acetate concentration in serum and feces of HFD-fed mice was significantly elevated after administration with BAA6. These results indicated that the increased levels of acetate were due to BAA6. It was well known that probiotic metabolites could play roles in regulating the expression of metabolism-related proteins [64]. Dietary SCFAs supplementation might enhance the proteins expression that related to FAO in adipose tissues [65]. Furthermore, the expression levels of these proteins were also elevated, after acetate treatment, in vivo and in vitro [44]. As expected, our data further confirmed that acetate was the necessary metabolite of BAA6 to enhance FAO.

Evidence has pointed out that the PPARα pathway has a primary action in the regulation of FAO [27]. Virtually, expression of most of the proteins during FAO processes was under the control of PPARα [66]. Rakhshandehroo et al. found that propionate up-regulated the PPARα expression in intestinal epithelial cells [66]. Our research obtained that acetate and BAA6-CM treatments could increase the protein expression of PPARα (by 16.51- and 3.84- fold) in vitro. Meanwhile, levels of PPARα protein were also enhanced, after BAA6 treatment, in HFD-fed mice. Multiple reports showed that deletion of PPARα would impair FAO in adipocytes of obese mice, resulting in fat accumulation [67]. In our study, acetate and BAA6-CM-induced enhancement of FAO were inhibited by PPARα siRNA. This result indicated that BAA6-generated acetate could regulate PPARα signaling to promote FAO. Besides, AMPK could mediate activation of PPARα signaling, which was the underlying mechanism for enhancement of FAO [45]. We observed that the levels of p-AMPK protein were significantly increased after acetate and BAA6-CM treatments. Furthermore, when 3T3-L1 cells were pretreated with Com (an inhibitor of AMPK), acetate and BAA6-CM-stimulated up-regulation of PPARα and FAO were diminished. Meanwhile, the levels of p-AMPK protein were also markedly increased after BAA6 supplementation in HFD-fed mice. These results proved that BAA6-generated acetate might activate AMPK-PPARα pathway signaling, which was associated with the up-regulation of FAO.

GPRs played an important role in cell signaling, especially in energy homeostasis, and regulated various adipocyte functions, such as FAO [21,23]. GPR43, as a key member of GPRs, might maintain the normal state of FAO through regulating related-genes expression [24,26]. Kimura et al. [68] reported that GPR43 knockout mice gained more weight compared with wild-type. Based on these, GPR43 has attracted attention as a potential therapeutic target for metabolic syndrome [69]. Low expression of GRP43 was found in the adipose tissues of obese mice, and dietary SCFAs supplementation significantly recovered its expression to decrease body weight [46]. As an important member of SCFAs, acetate could activate GPR43 signaling in adipocytes [70]. Consistently, in our study, acetate and BAA6-CM treatments could enhance the protein expression of GPR43 (by 181.55% and 131.78%) in vitro. Meanwhile, the levels of GPR43 protein were also remarkably raised in HFD-fed mice after administration with BAA6. In most cases, GPR43 is the main receptor of acetate [71]. Activation of GPR43 could regulate AMPK activity via Ca^2+^/CAMKKβ pathway [47]. Several reports found that the levels of intracellular Ca^2+^ and p-CAMKKβ were increased after treatment with the GPR43 agonist [72]. In line with these studies, our data showed that acetate and BAA6-CM treatments could elevate levels of Ca^2+^ (by 45.85% and 33.76%) and p-CAMKKβ (by 17.61- and 4.60- fold) in vitro. Besides, inhibition of GPR43 signaling abrogated expression of p-AMPK protein [73]. To further confirm the role of GPR43 on downstream signals, the expression of GPR43 in 3T3-L1 cells was silenced using GPR43 siRNA. Results showed that knockdown of GPR43 significantly prevented acetate and BAA6-CM-elicited up-regulation of intracellular Ca^2+^, p-CAMKKβ, p-AMPK, and PPARα. Moreover, acetate and BAA6-CM-induced enhancement of FAO were also destroyed by GPR43 knockdown. These findings indicated that BAA6-generated acetate could activate GPR43 signaling to promote FAO.

In conclusion, our study revealed that BAA6 supplementation could increase FAO in adipose tissues and alleviate the development of obesity. Acetate, an important metabolite of BAA6, was a pivotal factor to promote FAO through the GPR43-PPARα signaling. This study indicates that BAA6 may be a potential therapy to ameliorate diet-induced obesity. However, there are some limitations in our study. First, different age and sex of the mice need to be considered. In the future, clinical trials could be conducted to confirm the beneficial effect of BAA6 in obesity and obesity-related disorders.

## Data Availability

Data presented in this study are available on request from the corresponding author.

## Supplementary Materials

The following supporting information can be downloaded at: https://www.mdpi.com/article/10.3390/nu14030598/s1. Figure S1: Effect of BAA6 administration on lipid metabolism in HFD-fed mice, Figure S2: Effects of BAA6 culture supernatant (BAA6-CM) and dead BAA6 on cell viability in 3T3-L1 cells, Figure S3: Effect of dead BAA6 on TG concentration in 3T3-L1 cells, Figure S4: Effect of acetate or BAA6-CM on GPR41 expression levels of GPR41 in 3T3-L1 cells. Supplementary Table S1: Composition of diets, Supplementary Table S2: Small interfering RNA sequences applied in this experiment, Supplementary Table S3: Effect of oral administration of BAA6 on food intake in HFD-fed mice.
