# Supplementary material for: Bifidobacterium animalis subsp. lactis A6 Enhances Fatty Acid β-Oxidation of Adipose Tissue to Ameliorate the Development of Obesity in Mice
Source: Nutrients. 2022 Jan 29;14(3):598. doi: 10.3390/nu14030598 (PMC8839083; doi:10.3390/nu14030598)
Supplement: Supplementary file 1 [file nutrients-14-00598-s001.zip › Supplementary Figure S1, S2, S3, S4.pdf]

Supplementary Materials

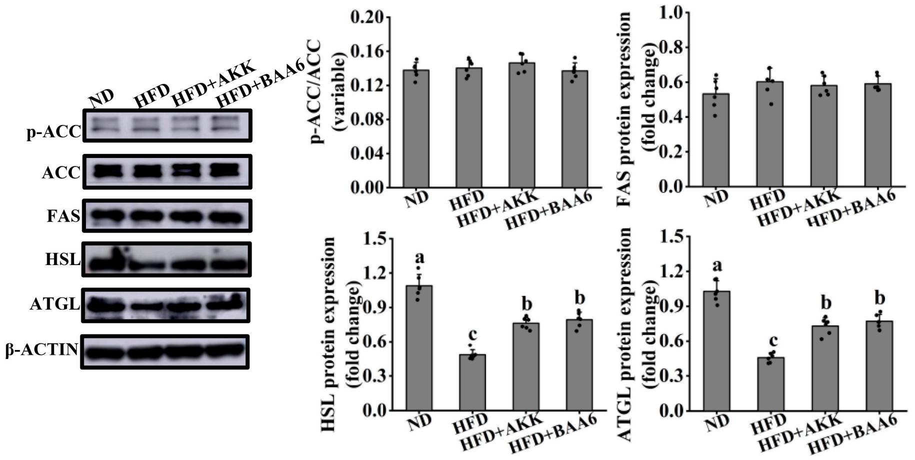

Figure S1. Effect of BAA6 administration on lipid metabolism in HFD-fed mice. Protein expression levels of p-ACC, FAS, HSL and ATGL following daily treatment with 10^9 colony-forming units (CFU)/kg of BAA6 or AKK. Values are expressed as means ± SD (n = 6). Different lowercase letters mean remarkable differences between groups at $p < 0.05$. HFD, fed high-fat diet for 17 weeks; HFD + BAA6, fed high-fat diet and treated with *Bifidobacterium animalis* subsp. *lactis* A6 (BAA6) for the final eight weeks; HFD + AKK, fed high-fat diet and treated with *Akkermansia muciniphila* (AKK) for the final eight weeks; ND, fed normal diet for 17 weeks; p-ACC, phosphorylated acetyl CoA carboxylase; FAS, fatty acid synthase; HSL, hormone-sensitive lipase; ATGL, adipose triglyceride lipase.

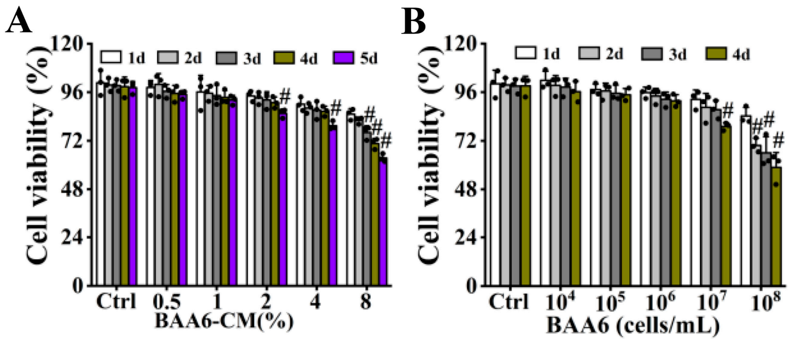

Figure S2. Effects of BAA6 culture supernatant (BAA6-CM) and dead BAA6 on cell viability in 3T3-L1 cells. (A) 3T3-L1 cells treated with BAA6-CM at different volume concentrations of the media (0-8%) for 1-5 d, respectively, as measured using cell counting kit-8 (CCK8) kit. (**B**) 3T3-L1 cells treated with dead BAA6 at different concentrations (0, 10^4, 10^5, 10^6, 10^7 and 10^8 cells/mL) for 1-4 d, respectively, as measured using CCK8 kit. Values are expressed as means ± SD. #*p* < 0.05 vs. different concentrations BAA6-CM or dead BAA6-treated cells at 1 d.

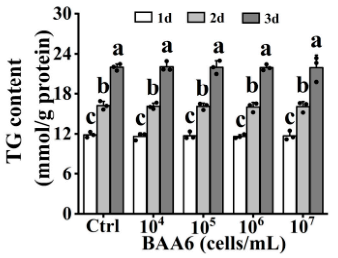

Figure S3. Effect of dead BAA6 (0-10^7 cells/mL) on TG concentrition in 3T3-L1 cells for 1-3 d. Values are expressed as means ± SD. Different lowercase letters mean remarkable differences between groups at $p < 0.05$. TG, triglyceride.

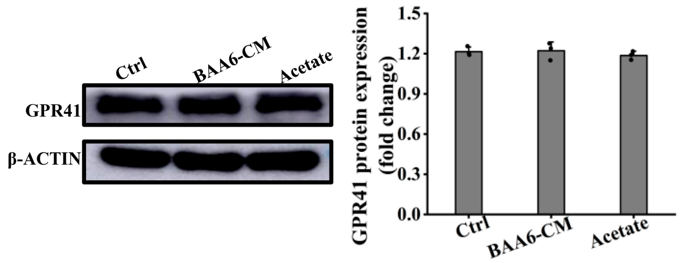

Figure S4. Effect of acetate or BAA6-CM on protein expression levels of GPR41 in 3T3-L1 cells. Western blot analysis showing the levels of GPR41 in 3T3-L1 cells after treatment with 4% BAA6-CM or 23.96 mmol/L acetate for 3 d. Values are expressed as means ± SD. GPR41, G protein-coupled receptor 41.
