# Supplementary material for: Bifidobacterium animalis subsp. lactis A6 Enhances Fatty Acid β-Oxidation of Adipose Tissue to Ameliorate the Development of Obesity in Mice
Source: Nutrients. 2022 Jan 29;14(3):598. doi: 10.3390/nu14030598 (PMC8839083; doi:10.3390/nu14030598)
Supplement: Supplementary file 1 [file nutrients-14-00598-s001.zip › Supplementary Table.pdf]

Supplementary Table S1. Composition of diets.

	ND		HFD	
Ingredient	gm	kcal	gm	kcal
Casein, 30 Mesh	200	800	200	800
L-Cystine	3	12	3	12
Corn Starch	315	1260	0	0
Maltodextrin 10	35	140	125	500
Sucrose	350	1400	68.8	275.2
Cellulose, BW200	50	0	50	0
Soybean Oil	25	225	25	225
Lard*	20	180	245	2205
Mineral Mix S10026	10	0	10	0
DiCalcium Phosphate	13	0	13	0
Calcium Carbonate	5.5	0	5.5	0
Potassium Citrate, 1 H2O	16.5	0	16.5	0
Vitamin Mix V10001	10	40	10	40
Choline Bitartrate	2	0	2	0
FD&C Yellow Dye #5	0.05	0	0.05	0
Total	1055.05	4057	773.85	4057
Content				
	gm%	kcal%	gm%	kcal%
Protein	19.2	20	26.2	20
Carbohydrate	67.3	70	26.3	20
Fat	4.3	10	34.9	60
Total		100		100
kcal/gm	3.85		5.24	

Supplementary Table S2. Small interfering RNA sequences applied in this experiment.

	Forward primer (5'→3')	Reverse primer (5'→3')
si*Pparα*	GCUGAGGAGCUAUUAUCCUUA	UAAGGCAUUGAACUUCAUAGC
si*Gpr43*	CCGGCCACUGUAUGGAGUGAU	AUCACUCCAUACAGUGGCCGG
sicon	UUCUCCGAACGUGUCACGUTT	ACGUGACACGUUCGGAGAATT

Supplementary Table S3. Effect of oral administration of BAA6 on food intake in HFD-fed mice[1]

	Food intake, g			
Time, weeks	ND	HFD	HFD+AKK	HFD+BAA6
1	17.57 ± 1.27	14.16 ± 1.20*	14.09 ± 1.20	14.19 ± 1.23
2	19.15 ± 1.14	17.04 ± 1.08*	17.05 ± 1.85	17.13 ± 0.92
3	20.44 ± 0.74	17.72 ± 1.34*	17.70 ± 1.13	17.75 ± 1.18
4	20.44 ± 1.31	18.01 ± 1.41*	17.95 ± 1.33	18.01 ± 1.20
5	20.95 ± 1.40	19.70 ± 1.78	19.40 ± 1.76	19.47 ± 1.35
6	21.04 ± 1.38	20.05 ± 1.31	19.62 ± 1.80	19.82 ± 1.47
7	21.11 ± 1.76	19.96 ± 1.16	19.41 ± 1.66	19.35 ± 1.81
8	20.99 ± 1.43	19.91 ± 1.44	19.38 ± 1.83	19.67 ± 1.54
9	21.08 ± 1.25	20.05 ± 1.40	19.60 ± 2.27	19.51 ± 1.78
10	20.89 ± 1.60	19.54± 1.74	19.49 ± 1.73	19.75 ± 2.70
11	21.05 ± 2.01	18.72± 2.19	18.95 ± 1.72	19.03± 2.76
12	20.77 ± 2.80	18.90 ± 2.13	18.76 ± 2.02	18.97 ± 2.01
13	20.87 ± 2.08	19.01 ± 2.48	18.87 ± 1.97	18.94 ± 2.05
14	21.16 ± 1.79	19.21 ± 2.08	19.10 ± 1.88	19.32 ± 1.91
15	20.94 ± 1.61	19.25 ± 2.12	18.81 ± 2.32	19.37 ± 2.05
16	21.05 ± 1.13	19.33 ± 2.21	19.01 ± 2.41	19.15 ± 1.75
17	21.20 ± 1.82	19.71 ± 2.17	19.34 ± 2.59	19.47 ± 1.58

[1] Values are expressed as means ± SD (n = 6, respectively). *$p < 0.05$ vs. ND group. HFD, fed high fat diet for 17 weeks; HFD+BAA6, fed high fat diet and the final 8 weeks treated with *Bifidobacterium animalis* subsp. *Lactis* A6 (BAA6); HFD+AKK, fed high fat diet and the final 8 weeks treated with *Akkermansia muciniphila* (AKK); ND, fed normal diet for 17 weeks.
